# Diagnosis of Aortoenteric Fistula in a 65‐Year‐Old Male With Recently Discovered Dieulafoy Lesion—A Case Report

**DOI:** 10.1155/crcc/1513556

**Published:** 2026-09-03

**Authors:** Amira Elsabagh, Mustafa Naveed, Luke Durbin, Thomas S. Davis, Mayada Issa, Yousuf M. Khan

**Affiliations:** ^1^ Virginia Tech Carilion Clinic Internal Medicine Residency, Roanoke, Virginia, USA; ^2^ Virginia Tech Carilion Clinic Internal Medicine Department, Roanoke, Virginia, USA

**Keywords:** abdominal, aorta, digestive system fistula, gastrointestinal hemorrhage, vascular grafting

## Abstract

**Background:**

Aortoenteric fistulas (AEFs) are an uncommon but often life‐threatening cause of gastrointestinal (GI) hemorrhage in patients with a history of abdominal aortic aneurysm (AAA) repair. Dieulafoy lesions are a rare cause of GI bleeding, which can have significant morbidity if not identified promptly.

**Case Report:**

In this case, we describe a 65‐year‐old male with a history of AAA repair, complicated by graft infection, who was admitted for GI bleeding. An initial esophagogastroduodenoscopy (EGD) revealed a Dieulafoy lesion in the gastric fundus, which was treated with endoscopic clips. The patient was discharged after clinical improvement. Two weeks later, he returned with recurrent bleeding and hemodynamic instability. Repeat EGD showed no active bleeding but revealed persistent blood in the stomach and duodenum. Subsequent imaging raised concern for AEF; ultimately, emergent surgical exploration confirmed the diagnosis, revealing graft dehiscence and communication into the duodenum.

**Conclusion:**

This case highlights the diagnostic challenge of GI bleeding in patients with a history of AAA repair. AEFs may present with “herald bleeds” that mimic other causes of GI bleeding such as Dieulafoy lesions, delaying definitive diagnosis and treatment. Subtle radiologic findings, such as perigraft air, induration, or loss of normal aortic fat planes, should prompt urgent surgical evaluation, particularly in patients with a history of aortic instrumentation, infected grafts, or recurrent bleeding despite endoscopic therapy. High clinical suspicion, early CT angiography, and timely surgical consultation are critical to improving outcomes in these patients.

## 1. Introduction

Dieulafoy lesions are a rare cause of acute gastrointestinal (GI) bleeds, representing only 1%–2% of cases. These lesions involve large, tortuous submucosal vessels that erode through the overlying mucosa and can cause life‐threatening bleeding. These lesions may bleed intermittently and can be found in various locations in the GI tract [1–3]. Endoscopic evaluation can be difficult, as lesions are often obscured by adherent clots or overlooked when surrounding gastritis is mistakenly assumed to be the source [1, 4]. However, early detection and treatment can decrease mortality [4].

Aortoenteric fistulas (AEFs) can be a cause of catastrophic GI bleeding. They involve a direct connection between the aorta and the GI tract. AEFs are categorized either as primary, which occur without prior aortic surgery, or secondary, which arise as a complication of AAA repair [5]. Most AEFs involve the abdominal aorta and primarily fistulize to the duodenum [5]. Secondary aortoenteric fistulas (SAEFs) are seen in 0.4%–1.6% of patients following an AAA repair, but that risk increases to 12%–33% in patients with graft infection [6, 7].

## 2. Case Presentation

**Figure 1:**
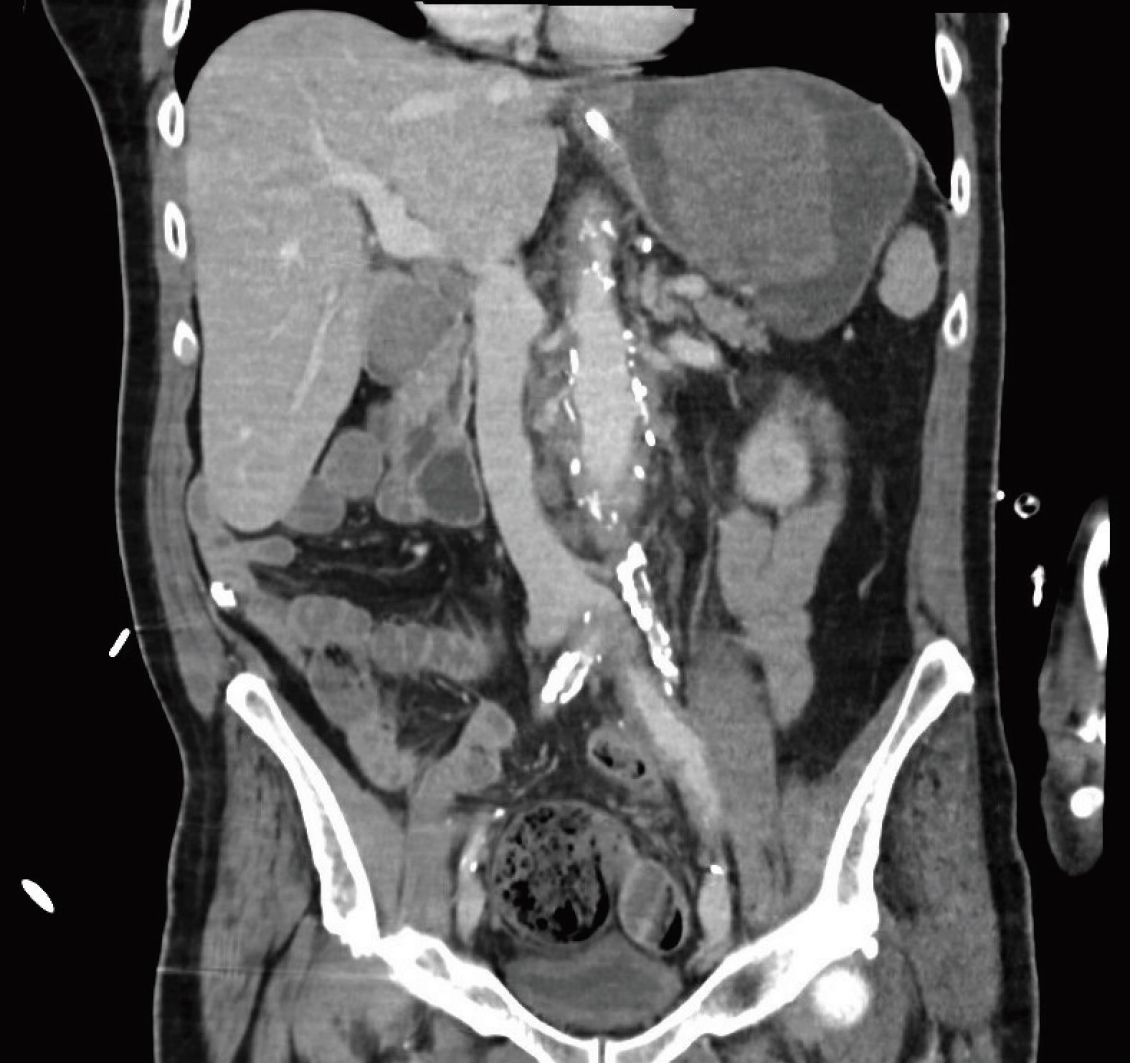
CTA of the abdomen and pelvis with large hyperdense intraluminal material in the stomach.

**Figure 2:**
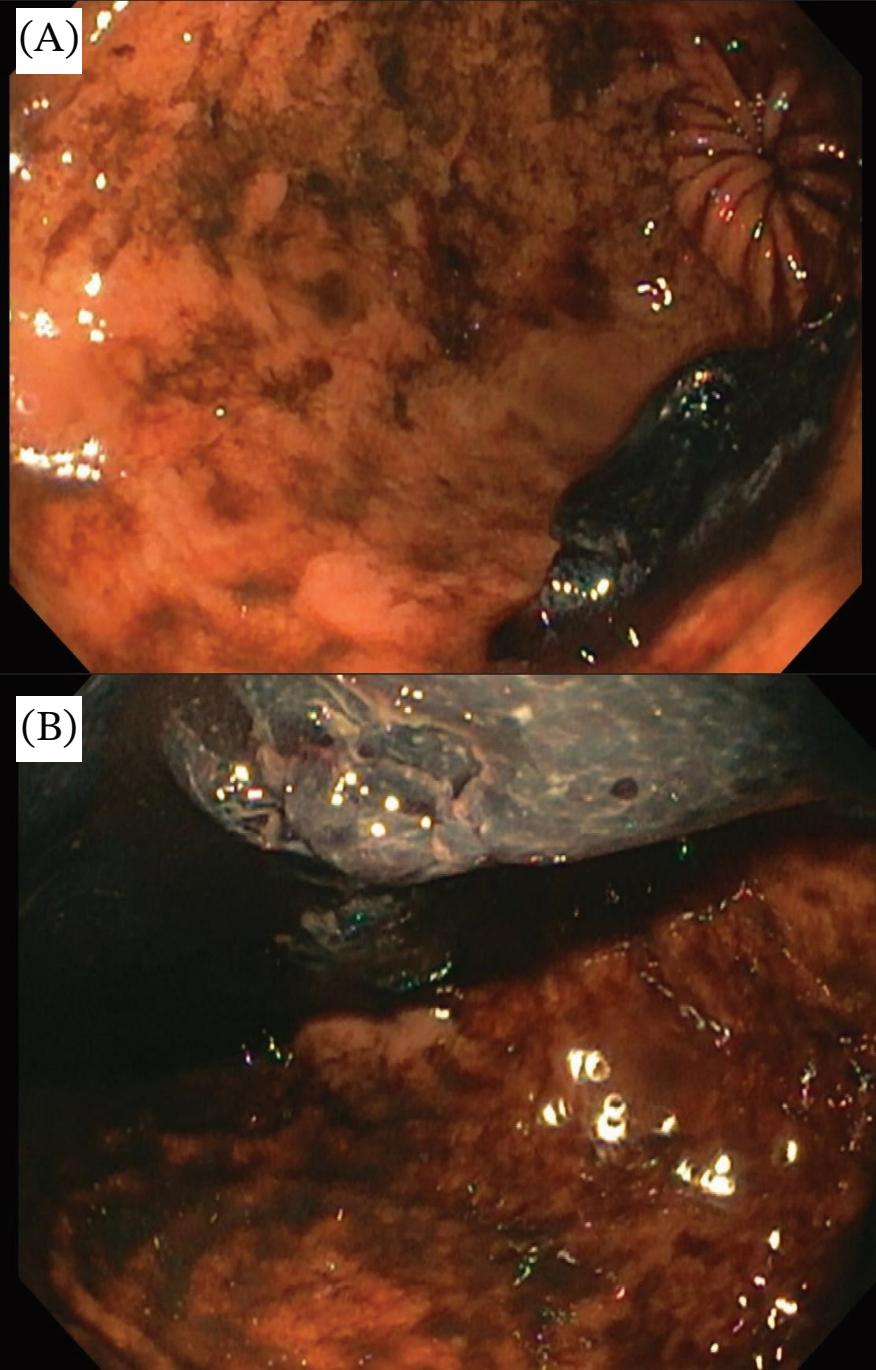
Endoscopic view of Dieulafoy lesion with adherent clot in the gastric fundus (A), beneath old blood products (B).

A 65‐year‐old male presented to the emergency department after a minor motor vehicle accident. The patient had a pertinent past medical history of abdominal aortic aneurysm status post aorta‐to‐right common iliac artery and left common femoral artery bypass with graft placement seven years prior, with a subsequent complication of graft infection status post placement of a rifampin‐soaked Dacron graft and peripheral arterial disease. Hours after this incident, the patient noted abdominal pain and lightheadedness and was later found significantly altered by his family after he developed hematemesis and rectal bleeding. He was intubated in the field by emergency medical services (EMS) for acute hypoxia as well as airway protection in the setting of active hematemesis. Initial work‐up was notable for a hemoglobin (Hgb) of 10.4 g/dL, platelets (PLTs) of 284 K/uL, prothrombin time (PT) of 13.0 s, international normalized ratio of 1.2, and activated partial thromboplastin time (aPTT) of 26.7 s. A computed tomography angiography (CTA) of the abdomen and pelvis revealed a large amount of intraluminal hyperdense material in the stomach, suspicious for bleeding (Figure 1). An orogastric tube was placed, yielding dark red output. The massive transfusion protocol (MTP) was initiated, and the patient was given six units of uncrossmatched packed red blood cells, six units of FFP, one unit of PLTs, and prothrombin complex concentrate. He was admitted to the ICU for hemorrhagic shock, which resolved with the transfusion, and he underwent an emergent esophagogastroduodenoscopy (EGD). The procedure revealed a Dieulafoy lesion with an adherent clot in the gastric fundus, beneath a significant amount of old blood products (Figure 2). Two endoscopic clips were placed at that time. Given the extent of his GI bleeding, rivaroxaban, which was held during admission, was discontinued. The patient was later started on 81 mg of aspirin (ASA). His Hgb remained stable, and the patient was discharged in stable condition two days after ASA initiation.

**Figure 3:**
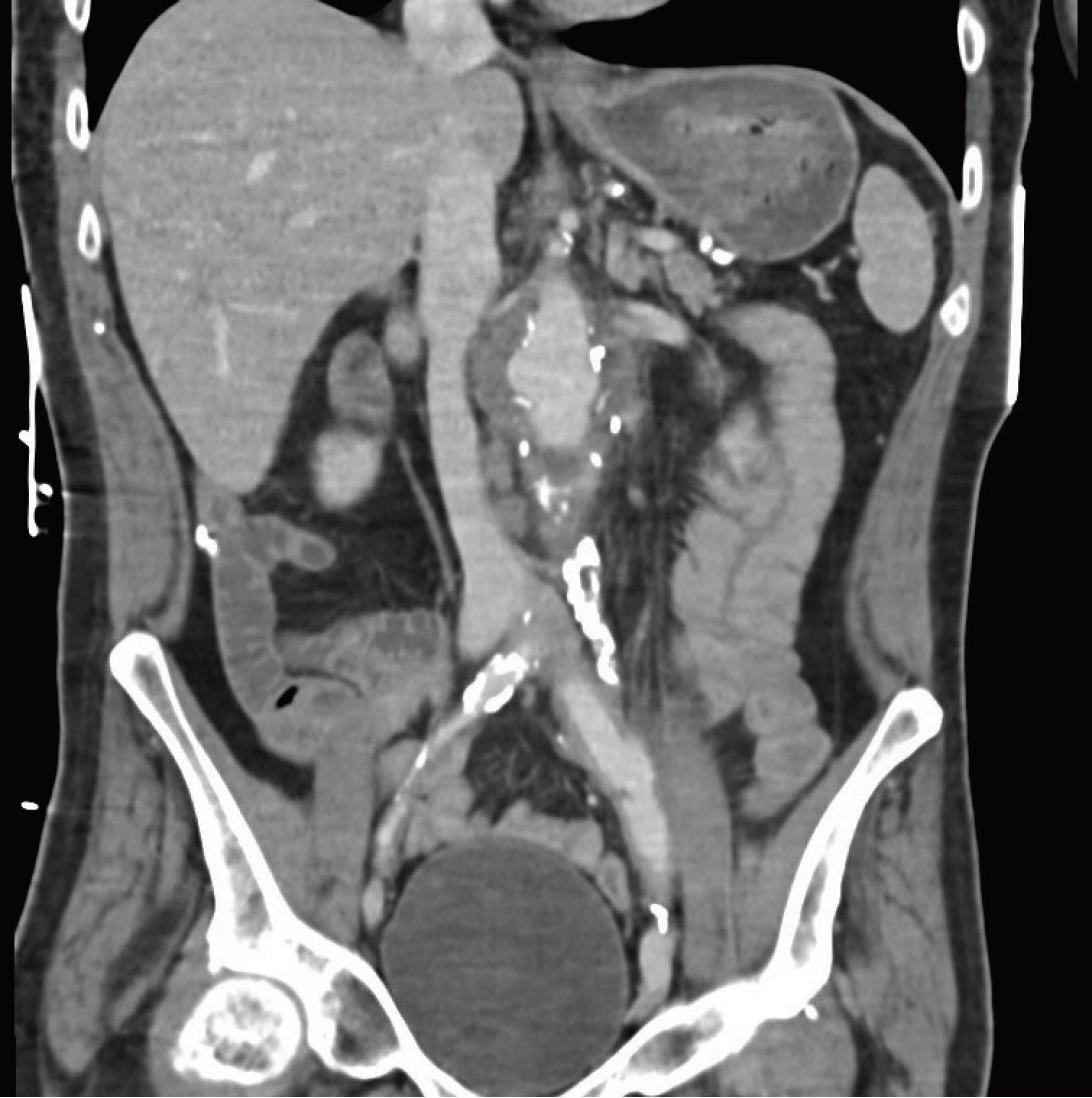
CTA of the abdomen and pelvis that shows slight increase in periaortic induration without evidence of contrast pooling or active bleeding.

**Figure 4:**
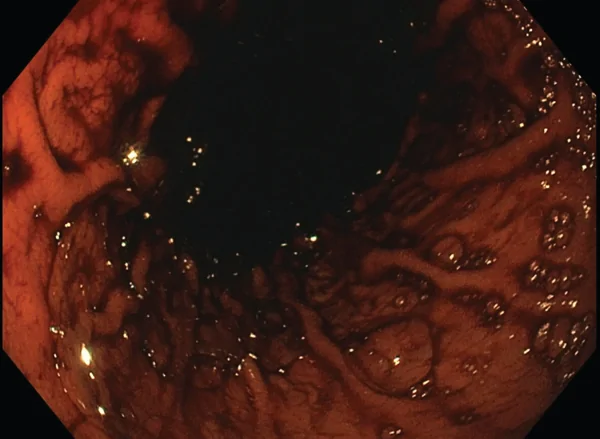
Endoscopic evidence of fresh maroon/black blood with dependent clots throughout the duodenum and stomach.

Approximately two weeks after discharge, he returned to the ER after being found unresponsive on the floor by his family. The patient became alert with EMS, and he stated that he had started having melena shortly after discharge and, the night prior, had experienced a significant amount of hematochezia. The patient had not continued rivaroxaban or ASA since his discharge. The labs were significant for a Hgb of 9.0 g/dL (down from 15.3 g/dL at discharge), PLTs of 499 K/uL, PT of 14.5 s, INR of 1.4, and a markedly elevated lactic acid of 18.5 mmol/L. A CTA of the abdomen and pelvis showed a slight increase in periaortic induration without evidence of contrast pooling or active bleeding (Figure 3). Gastroenterology was consulted for emergent EGD. The procedure revealed fresh maroon/black blood with dependent clots throughout the duodenum and stomach (Figure 4). In the fundus and cardia, the previous clips were found in place, and an additional two clips were deployed in close apposition.

The patient′s lactic acid normalized and Hgb remained stable overnight. Two days later, the patient had a repeat episode of melena, prompting an EGD extending to the third portion of the duodenum. There was no evidence of active bleeding, but an 8‐mm duodenal polyp was identified and removed via cold snare, with closure of the defect using two additional clips. The four clips previously placed were also visualized and remained stable at the site of the prior Dieulafoy lesion.

**Figure 5:**
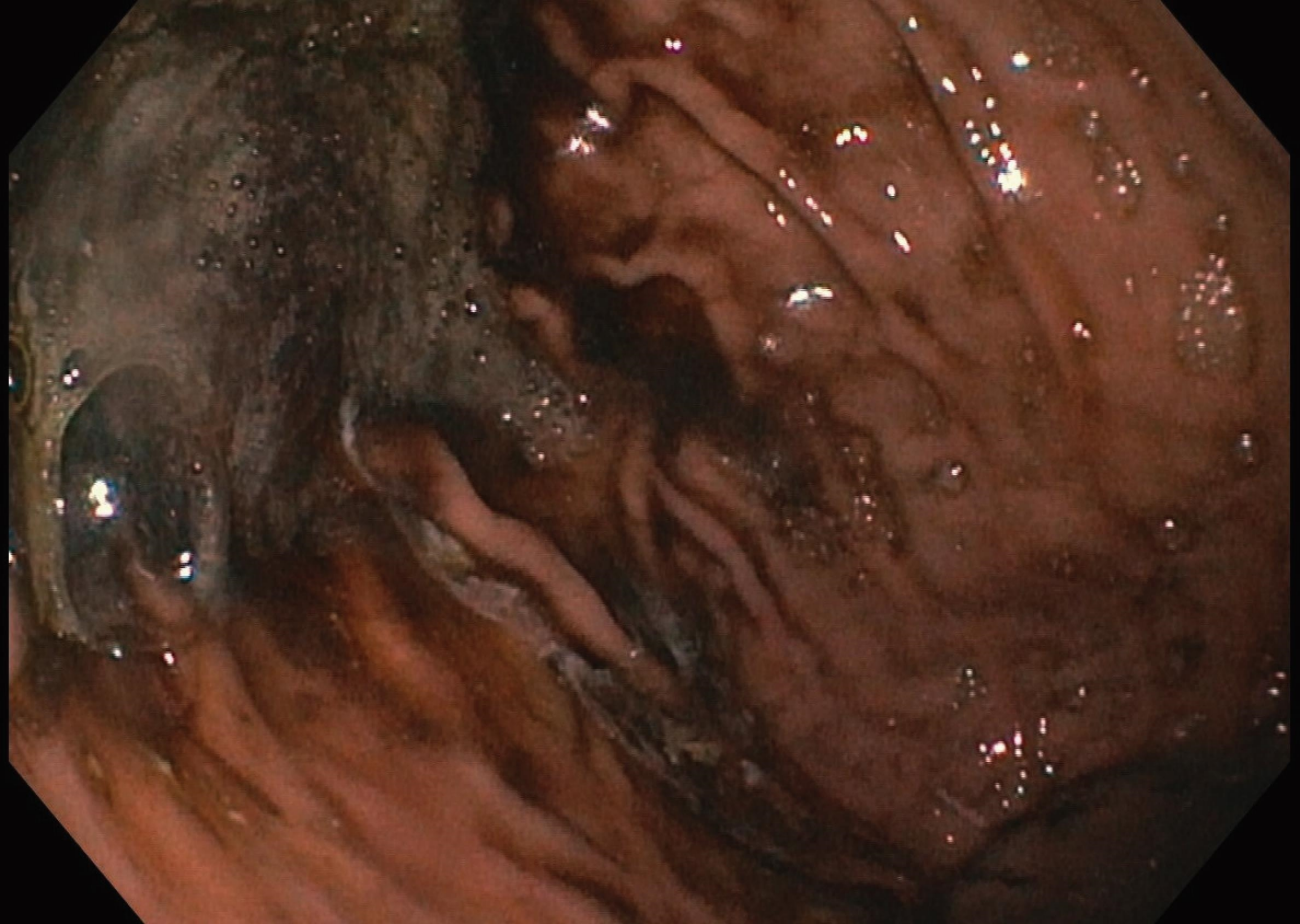
Repeat EGD demonstrated large blood clots at the previous site of the Dieulafoy lesion without an actively bleeding vessel.

**Figure 6:**
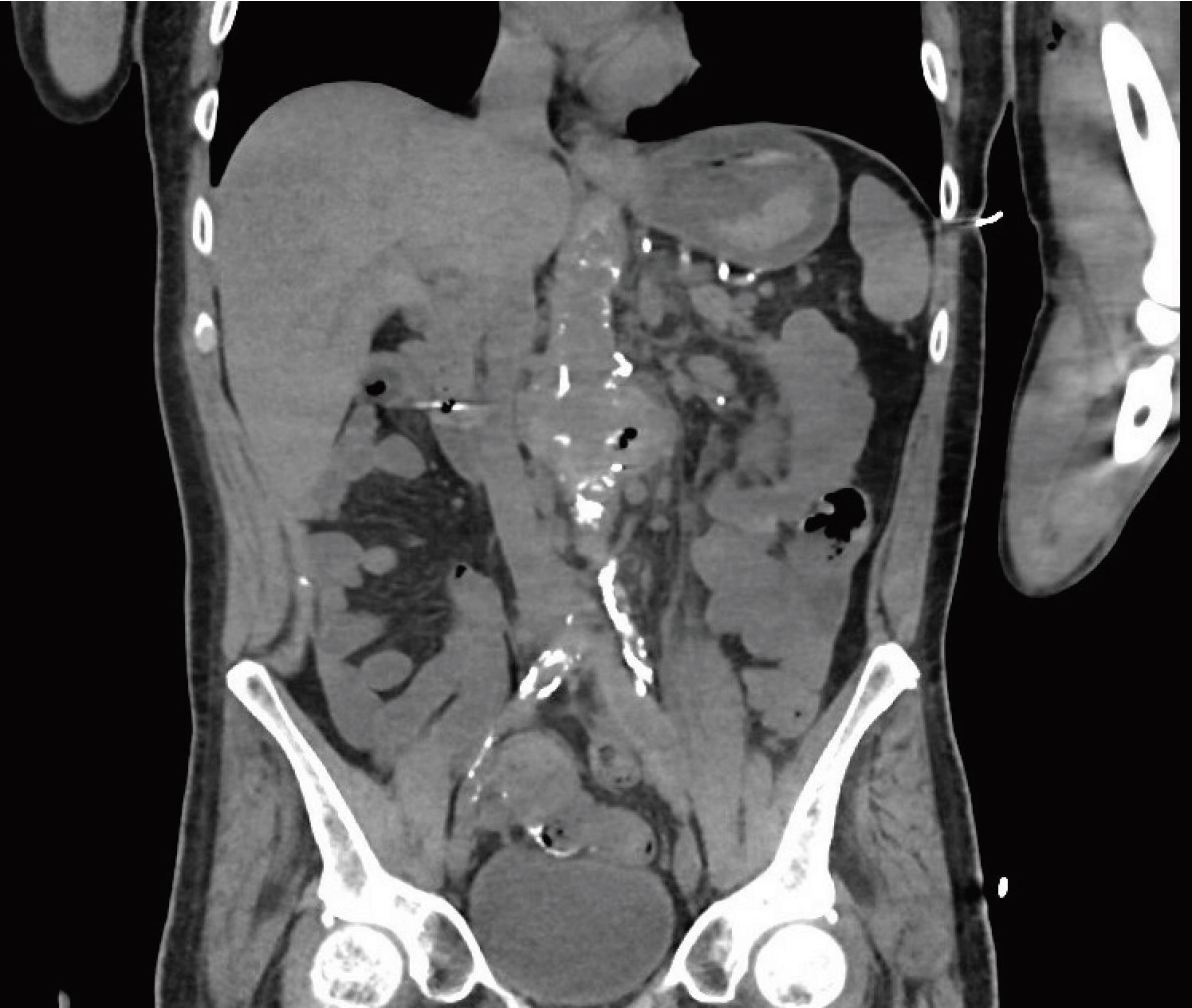
Repeat CTA of the abdomen and pelvis showed increased induration around the proximal graft along the mid‐aorta, with new findings of perigraft and a small area of contrast pooling anterior to the top of the graft.

The patient remained hemodynamically stable in the following days but complained of persistent lower back pain with paraspinal lumbar tenderness. Given the absence of active bleeding and his history of significant vasculopathy, the patient was restarted on ASA. Hgb improved until hospital Day 3, at which time it decreased from 11.1 to 8.6 g/dL. There were no clinical signs of bleeding, and the patient remained hemodynamically stable; ASA was discontinued. The Hgb continued to decline overnight, and the patient became hemodynamically unstable and was transferred to the ICU. Hgb was decreased to 7.0 g/dL, PT was elevated to 16.2 s, INR 1.6, and aPTT was stable at 26.5 s. The patient developed large‐volume maroon stools and became persistently hypotensive despite fluid resuscitation and three units of pRBCs. An emergent repeat EGD demonstrated large blood clots at the previous site of the Dieulafoy lesion without an actively bleeding vessel (Figure 5). Two additional clips were placed in the area of mucosal friability. A repeat CTA of the abdomen and pelvis showed increased induration around the proximal graft along the mid‐aorta, with new findings of a small area of contrast pooling anterior to the top of the graft as well as perigraft air (Figure 6). Vasopressor support with norepinephrine and vasopressin was initiated, and the MTP was reactivated. Vascular surgery was consulted for emergent surgical exploration due to concern for an AEF.

The patient was taken urgently to the operating room. The viscera were retracted, and dissection was performed down to the aortic graft. The aorta in this area was opened into what appeared to be a phlegmon around the poorly incorporated graft beyond the bifurcation. Further dissection revealed dehiscence of the anastomosis with bleeding into the duodenum due to an AEF. General surgery was consulted intraoperatively for assistance with temporary enterotomy closure. The infected graft was removed and replaced with a cadaveric bypass graft from the proximal aorta to both femoral arteries. The patient returned to the ICU with a temporary abdominal wall closure and open abdominal negative‐pressure wound therapy, with plans for repeat exploration within 24 h.

Upon the patient′s arrival in the ICU from the OR, Hgb was found to be 5.7 g/dL, PLTs PT were 16.9 s, aPTT was elevated to 78.3, and ROTEM was significantly irregular, most notable for extended clotting time and decreased alpha angle. These results were suggestive of a disseminated coagulopathy. Lactic acid increased from 0.6 to 15.7 mmol/L. Despite aggressive resuscitation and reversal of coagulopathy with three rounds of the MTP, the patient continued to have increased sanguineous output from the drain. Given these findings and family discussions with the surgical team, the family elected to transition to comfort‐focused care. The patient was extubated, and the time of death was called approximately 15 min later.

## 3. Discussion

In this case, we describe a patient presenting with GI bleeding secondary to a recently diagnosed Dieulafoy lesion, who was subsequently found to have developed a SAEF.

SAEFs, as seen in this case, are a rare cause of GI bleeding and occur as a complication of prior aortic surgery or graft placement, often when complicated by a graft infection. The infection may lead to weakening of the arterial wall and the formation of a pseudoaneurysm causing pulsatile shearing against the GI mucosa [5]. The most common presentation is GI bleeding; it may present as a herald bleed or hemorrhagic shock, and rarely can include back pain, pseudoaneurysms, and limb ischemia [5, 8]. Herald bleeds, defined as transient, self‐limited episodes of hematemesis, melena, or hematochezia, occur in up to 80% of presentations. The self‐limitation is thought to be due to thrombus formation or contraction of the intestinal wall surrounding the fistula [5, 7, 8]. A scholarly review reveals that the duration of time between the herald bleed and the severe bleeding varies from a few hours to several months, with an average of 3–4 days [5, 7].

In the presented patient, diagnosing the AEF was challenging because he had previously been diagnosed with a Dieulafoy lesion, a plausible alternative cause of recurrent, large‐volume arterial bleeding. Repeated endoscopic evaluation revealed multiple clots at the site of the initial aberrant vasculature, making this lesion the suspected primary source. Evaluation in the setting of large‐volume GI bleeds often involves endoscopy. However, in cases such as Bhatti et al. [9], endoscopic evaluation did not yield any sign of intraluminal GI bleeding in the setting of an AEF. EGD evaluation has limited utility because, although most fistulas involve the duodenum, they typically occur in the third to fourth portion, which requires enteroscopy, not available in all facilities. The utility of EGD is limited, showing clinical findings in only 40%–50% of cases and being diagnostic for AEFs in only 25%–40%. It provides a measure for ruling out other causes of GI bleeding [5, 7–9].

Our patient′s history of aortic instrumentation, previously infected aortic graft and the recurrent GI bleeding should raise clinical concern for development of SAEFs as an alternative cause. The mainstay for diagnosis is the CT with a sensitivity of approximately 90%–95% [5]. In this case, initially performed CTA did reveal some increased induration surrounding the graft site, but it was not apparent until repeat imaging with the patient′s clinical decompensation that showed perigraft air. Retrospective analysis emphasizes the diagnostic value of subtle radiographic signs such as perigraft soft‐tissue edema, fluid, ectopic gas, or hematoma. Other radiologic cues, including pseudoaneurysm formation or disruption of the aortic wall, should also raise suspicion for fistulization [10].

Definitive management of AEFs requires urgent surgery and if untreated, the mortality is 100% [5, 8, 9]. Treatment often involves removal of the infected graft, bowel repair, and either in situ graft replacement or extra‐anatomic bypass. Endovascular aortic repair (EVAR) may be used as a temporizing measure in unstable patients, offering rapid hemorrhage control, but it does not address the enteric defect or infection. Open surgical repair remains the standard of care should be individualized based on patient stability and infection burden [11].

In patients with significant GI bleeding and a history aortic instrumentation, clinicians must maintain a high index of suspicion for AEF. Prompt imaging with CTA and timely surgical consultation are critical to improve outcomes in this potentially fatal condition.

## Funding

No funding was received for this manuscript.

## Consent

No written consent was obtained from the patient, as there is no patient‐identifiable data included in this case report.

## Conflicts of Interest

The authors declare no conflicts of interest.

## Supporting information


**Supporting Information** Additional supporting information can be found online in the Supporting Information section. This case report was prepared in accordance with CARE guidelines.

## Data Availability

Data sharing not applicable to this article as no datasets were generated or analysed during the current study.
