# Supplementary material for: Diagnosis of Aortoenteric Fistula in a 65‐Year‐Old Male With Recently Discovered Dieulafoy Lesion—A Case Report
Source: Case Rep Crit Care. 2026 Sep 3;2026:1513556. doi: 10.1155/crcc/1513556 (PMC13542581; doi:10.1155/crcc/1513556)
Supplement: Supplementary file 1 [file CRCC-2026-1513556-s001.pdf]

CARE case report guidelines

CARE Checklist of information to include when writing a case report

(cc) BY-NC-ND

Topic	Item	Checklist item description	Reported on Line
Title	1	The diagnosis or intervention of primary focus followed by the words "case report"	Title Page
Key Words	2	2 to 5 key words that identify diagnoses or interventions in this case report, including "case report"	155-156
Abstract (no references)	3a	Introduction: What is unique about this case and what does it add to the scientific literature?	6-8
	3b	Main symptoms and/or important clinical findings	11
	3c	The main diagnoses, therapeutic interventions, and outcomes	14-16
	3d	Conclusion—What is the main "take-away" lesson(s) from this case?	18-24
Introduction	4	One or two paragraphs summarizing why this case is unique (**may include references**)	26-37
Patient Information	5a	De-identified patient specific information.	40-44
	5b	Primary concerns and symptoms of the patient.	45-46
	5c	Medical, family, and psycho-social history including relevant genetic information	41-44
	5d	Relevant past interventions with outcomes	52-59
Clinical Findings	6	Describe significant physical examination (PE) and important clinical findings.	61-66
Timeline	7	Historical and current information from this episode of care organized as a timeline	67-105
Diagnostic Assessment	8a	Diagnostic testing (such as PE, laboratory testing, imaging, surveys).	Throughout
	8b	Diagnostic challenges (such as access to testing, financial, or cultural)	N/A
	8c	Diagnosis (including other diagnoses considered)	90
	8d	Prognosis (such as staging in oncology) where applicable	N/A
Therapeutic Intervention	9a	Types of therapeutic intervention (such as pharmacologic, surgical, preventive, self-care)	endoscopic, surgical
	9b	Administration of therapeutic intervention (such as dosage, strength, duration)	N/A
	9c	Changes in therapeutic intervention (with rationale)	Throughout
Follow-up and Outcomes	10a	Clinician and patient-assessed outcomes (if available)	N/A
	10b	Important follow-up diagnostic and other test results	Throughout
	10c	Intervention adherence and tolerability (How was this assessed?)	N/A
	10d	Adverse and unanticipated events	101-105
Discussion	11a	A scientific discussion of the strengths AND limitations associated with this case report	120-137
	11b	Discussion of the relevant medical literature **with references**.	108-143
	11c	The scientific rationale for any conclusions (including assessment of possible causes)	52-59
	11d	The primary "take-away" lessons of this case report (without references) in a one paragraph conclusion	144-146
Patient Perspective	12	The patient should share their perspective in one to two paragraphs on the treatment(s) they received	N/A, patient deceased
Informed Consent	13	Did the patient give informed consent? Please provide if requested	Yes ■ No ☐
